# The inhibition of FKBP5 protects β-cell survival under inflammation stress via AKT/FOXO1 signaling

**DOI:** 10.1038/s41420-023-01506-x

**Published:** 2023-07-14

**Authors:** Na Liu, Rui Li, Jinglin Cao, Xinyao Song, Wenmiao Ma, Tengli Liu, Le Wang, Jiaqi Zou, Boya Zhang, Zewen Liu, Rui Liang, Rongxiu Zheng, Shusen Wang

**Affiliations:** 1 Department of Pediatrics, Tianjin Medical University General Hospital, 300052 Tianjin, People’s Republic of China; 2 Research Institute of Transplant Medicine, Organ Transplant Center, NHC Key Laboratory for Critical Care Medicine, Tianjin First Central Hospital, Nankai University, 300384 Tianjin, People’s Republic of China; 3 Department of Hepatobiliary Surgery, The Third Hospital of Hebei Medical University, 050051 Shijiazhuang, People’s Republic of China

**Keywords:** Type 2 diabetes, Calcium and phosphate metabolic disorders

## Abstract

The FK506-binding protein 51 (FKBP51, encoded by *FKBP5* gene) has emerged as a critical regulator of mammalian endocrine stress responses and as a potential pharmacological target for metabolic disorders, including type 2 diabetes (T2D). However, in β cells, which secrete the only glucose-lowering hormone—insulin, the expression and function of FKBP5 has not been documented. Here, using human pancreatic tissue and primary human islets, we demonstrated the abundant expression of FKBP5 in β cells, which displayed an responsive induction upon acute inflammatory stress mimicked by in vitro treatment with a cocktail of inflammatory cytokines (IL-1β, IFN-γ, and TNF-α). To explore its function, siRNAs targeting FKBP5 and pharmacological inhibitor SAFit2 were applied both in clonal NIT-1 cells and primary human/mice islets. We found that FKBP5 inhibition promoted β-cell survival, improved insulin secretion, and upregulated β-cell functional gene expressions (*MAFA* and *NKX6.1*) in acute-inflammation stressed β cells. In primary human and mice islets, which constitutively suffer from inflammation stress during isolation and culture, FKBP5 inhibition also presented decent performance in improving islet function, in accordance with its protective effect against inflammation. Molecular studies found that FKBP5 is an important regulator for FOXO1 phosphorylation at Serine 256, and silencing of FOXO1 abrogated the protective effect of FKBP5 inhibition, suggesting that it is the key downstream effector of FKBP5 in β cells. At last, in situ detection of FKBP5 protein expression on human and mice pancreases revealed a reduction of FKBP5 expression in β cells in human T2D patients, as well as T2D mice model (db/db), which may indicate a FKBP5-inhibition-mediated pro-survival mechanism against the complex stresses in T2D milieus.

## Highlights

- The FK506-binding protein 51 is expressed in human β cells.
- FKBP5 inhibition protects β-cell survival under inflammation stress.
- FOXO1 is the key downstream effector to mediate FKBP5 functioning in β cells.
- FKBP5 is downregulated in T2D, which may indicate a FKBP5-inhibition-mediated pro-survival mechanism.

## Introduction

More than 400 million people suffers from diabetes and its complications worldwide, resulting in a growing burden on public health [1]. Among them over 90% are type 2 diabetes (T2D), manifested by peripheral insulin resistance and inadequate insulin secretion. Glucose toxicity, lipid toxicity, and inflammation are critical factors that influence insulin secretion either by impairing β-cell function or causing functional β-cell mass loss [2, 3]. A better understanding of the molecular mechanisms of β-cell dysfunction in T2D progression is essential to develop new therapies to preserve β-cell function.

The FK506-binding protein 51 (FKBP51, encoded by the *FKBP5* gene) belongs to immunophilin class proteins due to its activity of peptidyl-prolyl cis-trans isomerase (PPIase) that is inhibited by immunosuppressant ligands, such as FK506 and rapamycin [4]. Meanwhile, FKBP5 also has a tetratricopeptide repeat (TPR) motif that functions to bind diverse client proteins via protein-protein interaction. Recently, accumulating evidence suggests that FKBP5 plays as a key regulator in obesity and diabetes. FKBP5 serves as a cochaperone to inhibit glucocorticoid receptor (GR) induced lipolysis of stored lipids in white adipose tissue and to promote peroxisome-activated receptor γ (PPARγ) induced adipogenesis and lipid storage in adipocytes [5–7]. Complete loss of FKBP5 in mice model shows improved glucose tolerance, which suggests FKBP5 is involved in glucose homeostasis [8, 9]. Although its role in adipogenesis has been indicated in the peripheral insulin resistance during T2D development, the function of FKBP5 in islet, the central regulator of glucose metabolism, remains unknown.

In this study, we discovered the expression of FKBP5 in β cells and characterized its expression change during acute-inflammation stress in vitro and the long-term diseased condition in T2D. FKBP5 inhibition promotes β-cell survival in acute-inflammation stress and hence reserving β-cell function. FKBP5 is an important regulator for FOXO1 by regulating its phosphorylation state; meanwhile, FOXO1 is a critical mediator for FKBP5 functioning in β cells. In addition, reduced expression of FKBP5 in β cells of T2D patients may indicate a FKBP5-inhibition-mediated pro-survival mechanism against the complex stresses in type 2 diabetic milieus.

## Results

### Inflammation-induced FKBP5 expression in human islets, mice islets, and clonal β cells

**Fig. 1:**
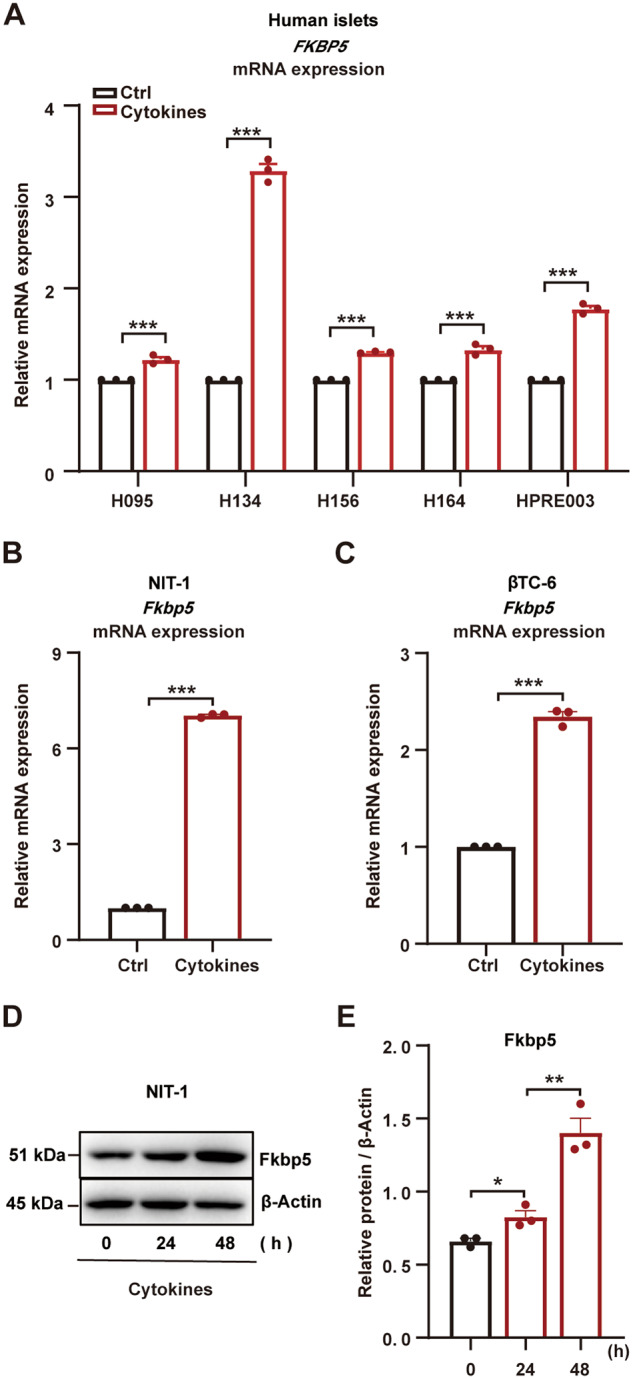
Proinflammatory cytokines induced FKBP5 expression in human islets, mice islets, and clonal β cells. **A** mRNA expression of *FKBP5* in human islets from 5 organ donors treated with proinflammatory cytokines (IL-1β 10 ng/mL, TNF-α 25 ng/mL, IFN-γ 100 ng/mL). Experiments were repeated for 3 times. **B**, **C** mRNA expression of *Fkbp5* in NIT-1 cells (**B**) and βTC-6 cells (**C**). Experiments were repeated for 3 times. **D**, **E** Western blot of Fkbp5 in NIT-1 cells treated with proinflammatory cytokines (**D**) and Fkbp5 expression levels were quantified by Image J, with β-actin as loading control. **E** Experiments were repeated for 3 times. Student’s *t*-test. Mean ± SEM, *P＜0.05, **P＜0.01, ***P <0.001.

Local islet inflammation is a critical stress that causes β-cell dysfunction and mass loss [10, 11]. In this study, using inflammatory cytokines IL-1β (10 ng/mL), TNF-α (25 ng/mL), and IFN-γ (100 ng/mL) to mimic inflammatory stress, we found that *FKBP5* mRNA expression was significantly induced in human islets upon these cytokines treatment (Fig. 1A). Similarly, in clonal β cells NIT-1 and β-TC-6, cytokines treatment also significantly upregulated *Fkbp5* mRNA expression (Fig. 1B, C). Western blot assays confirmed the upregulation of Fkbp5 by inflammation treatment at protein level in NIT-1 cells (Fig. 1D, E). These results suggested that FKBP5 expression was upregulated in response to inflammatory stimulus.

### FKBP5 inhibition improves β-cell survival upon inflammation insult via autophagy regulation

**Fig. 2:**
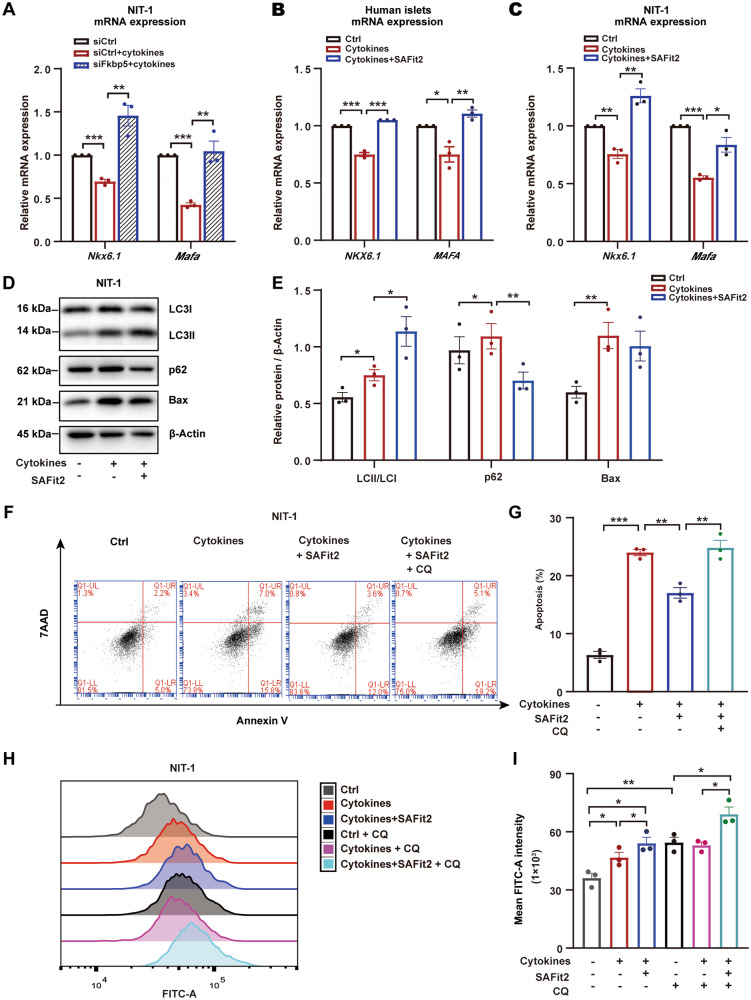
FKBP5 inhibition protected β cells against proinflammatory cytokines-induced apoptosis via autophagy regulation. **A** NIT-1 cells were transfected with siFkbp5 or control siRNAs and then treated with cytokines, mRNA expression of *Nkx6.1* and *Mafa* were evaluated by qRT-PCR. Experiments were repeated for 3 times. **B**, **C** mRNA expression of *Nkx6.1* and *Mafa* in NIT-1 cells (**B**) and primary human islets (**C**) treated with proinflammatory cytokines (Cytokines), or cytokines together with SAFit2 (Cytokines + SAFit2). Experiments were repeated for 3 times. **D**, **E** Western blot with LC3, p62 and Bax in the cytokines, or cytokines+ SAFit2-treated NIT-1 cells (**D**). The signal intensity was quantified by Image J with β-actin as loading control. Experiments were repeated for 3 times (**E**). **F**, **G** Annexin-V/7-AAD staining and flow cytometry analysis in NIT-1 cells treated with cytokines, cytokines + SAFit2, or Cytokines + SAFit2 + CQ) (**F**). Quantification of the apoptotic cell rate (PE^+^7AAD^+^/PE^+^7AAD^-^). Experiments were repeated for 3 times (**G**). **H**, **I** Autophagy level measurement by flow cytometry in NIT-1 cells treated with Cytokines, or Cytokines + SAFit2. CQ was used to allow the accumulation of autophagic vacuoles. FITC-A intensity represents the autophagy level, Experiments were repeated for 3 times.. Student’s *t*-test. Mean ± SEM, *P < 0.05, **P < 0.01, ***P < 0.001.

Using siRNAs to prevent the upregulation of Fkbp5 by inflammation, we found that although the inflammation treatment downregulated the mRNA expression level of *Nkx6.1*, *Mafa* in siCtrl cells, this effect was reversed in siFkbp5 cells (Fig. 2A). Similarly, pretreatment with SAFit2, a highly selective FKBP5 inhibitor via disrupting the scaffolding function of FKBP5, also abrogated the downregulation of *NKX6.1*, *MAFA* mRNA expression by inflammatory cytokines in human islet cells and NIT-1 cells (Fig. 2B, C). Together, these results confirmed that FKBP5 inhibition protects β-cell dysfunction against inflammation insult.

Then we asked whether FKBP5 played a role in β-cell survival under inflammatory stress. In NIT-1 cells, we found that the protein expression Bax, a proapoptotic marker, was significantly upregulated in inflammation treatment group, but it was restored in the presence of SAFit2 treatment (Fig. 2D, E), indicating that FKBP5 inhibition by SAFit2 may promote β-cell survival. To this end, cell apoptosis was assayed using Annexin-V/7-AAD staining followed by flow cytometry. The result demonstrated that inflammation cytokines treatment significantly increased the apoptotic rate of NIT-1 cells but SAFit2 pretreatment restored β-cell survival (Fig. 2F, G). Since autophagy is a fundamental survival mechanism for β cells under stresses, we further examined β-cell apoptosis induced by inflammation in the presence of a potent autophagy blocker, CQ. The results showed that the presence of CQ completely abrogated the protective effect of SAFit (Fig. 2F, G), indicating that autophagy may plays a role in mediating the protection of Fkbp5 inhibiton on β-cell survival under inflammation stress.

Therefore, we further explored whether FKBP5 regulates β-cell autophagy. The expression and localization of microtubule-associated protein 1 light chain 3 (LC3) and SQSTM1/p62 are most commonly used to address the levels of autophagy. Increased processing of LC3-I into LC3-II can be observed when stimulating autophagy activity and blocking autophagic flux. The Sqstm1 protein, as a link between LC3-II and ubiquitinated substrates, binding polyubiquitinated proteins become incorporated into the completed autophagosome and are degraded in autolysosomes, thus serving as an index of autophagic degradation [12]. The western blot results revealed that the expression of LC3-II accumulated in inflammation treatment group, and it was further upregulated in the presence of SAFit2. The expression of Sqstm1 increased in NIT-1 cells treated with inflammatory cytokines, but decreased in SAFit2 treatment group (Fig. 2D, E). Using CQ to allow the accumulation of autophagic vacuoles, with Cyto-ID staining to indicate the autophagic level and PI staining to exclude the dead cells, we found that the accumulation of autophagic vacuoles increased by CQ in control condition, while in NIT-1 cells treated with inflammatory cytokines, the autophagic level did not significantly increase in presence of CQ (Fig. 2H, I), suggesting that inflammation stress has already impaired the degradation and recycling process in autophagy flux, which was consistent with previous study that inflammation caused autophagy dysfunction in β cells [13]. In contrast, SAFit2 further elevated the accumulation of autophagic vacuoles of inflammation treated β cells in the presence of CQ (Fig. 2H, I), suggesting that SAFits restored the recycling of autophagosomes and expanded the autophagy capacity. Together, these results suggested that FKBP5 inhibition protected β-cell survival under inflammation stresses, which is probably mediated by autophagy level resotration.

### FKBP5 inhibition improves β-cell function in ex vivo-cultured mice and human islets

**Fig. 3:**
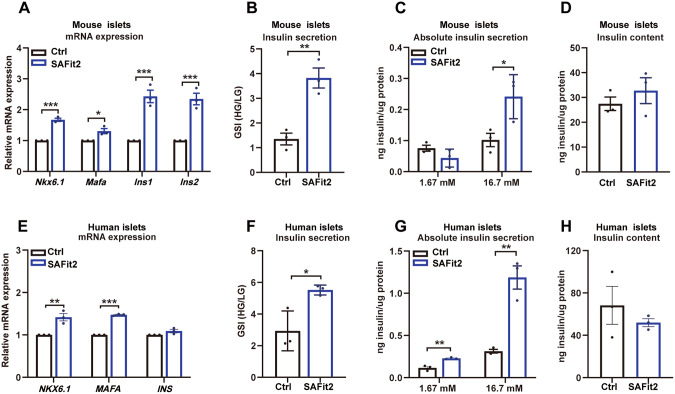
FKBP5 inhibition improved β-cell function in primary human and mice islets. **A** mRNA expression of *Nkx6.1, Mafa, Ins1, Ins*2 in primary mice islets treated with SAFit2 or control vehicle for 48 h. Experiments were repeated for 3 times. **B**, **D** GSI (**B**), insulin secretions (**C**) and intracellular insulin contents (**D**) in primary mice islets treated with SAFit2 or control vehicle for 48 h. Data were generated from three repeated experiments. **E** mRNA expression of *NKX6.1, MAFA, INS* in primary human islets treated with SAFit2 or control vehicle for 48 h. Experiments were repeated for 3 times. **F**, **H** GSI (**F**), insulin secretion (**G**) and intracellular insulin content (**H**) in primary human islets treated with SAFit2 or control vehicle for 48 h. Data were generated from three repeated experiments. Student’s *t*-test. Mean ± SEM, *P < 0.05, **P < 0.01, ***P < 0.001.

Since inflammation stress is also present in the isolation and ex vivo culture process of primary islet cell clusters, we examined whether FKBP5 plays a role in improving the function of primary islets. Using SAFit2 to inhibit the function of FKBP5 in ex vivo-cultured primary islets, we found that SAFit2 significantly improved the expression of β-cell functional gene expressions both in mice islets (*Nkx6.1, Mafa, Ins1, and ins2*, Fig. 3A) and human islets (*NKX6.1, MAFA, and INS*, Fig. 3E). Further glucose-stimulated insulin secretion (GSIS) assay results showed that SAFit2 significantly upregulated the glucose-stimulated insulin secretion both in human and mice islets, evidenced by the significantly higher glucose-stimulated index (GSI) (Fig. 3B, F) and higher absolute insulin secretion at high-glucose condition (Fig. 3C, G). No significant influence on insulin contents were observed (Fig. 3D, H). Similarly, knockdown FKBP5 using siRNAs also improved the functional gene expressions (Fig. S1A, C) and GSI (Fig. S1B, D) both in ex vivo-cultured mice and human primary islets. Previous studies on islet preparation have indicated that during islet isolation and culture, islets suffer from the stresses such as inflammation and hypoxia [14–16]. Therefore, these results together suggested that FKBP5 knockdown prevented the decay of islet function during ex vivo culture both in mice and human islets.

We further explore the impact of Fkbp5 knockdown on insulin secretion by direct inflammatory cytokines treatment. The GSIS results revealed that proinflammatory cytokines treatment significantly impaired the GSI (Fig. S2A), with uncontroled insulin secretion at low glucose, and significantly impaired the insulin secretion at high-glucose condition (Fig. S2B). Compared to the inflammation group, knockdown of Fkbp5 partially restored GSI (Fig. S2A) by inhibiting the insulin secretion at low-glucose condition and increasing the insulin secretion at high-glucose condition (Fig. S2B). Both inflammation cytokines treatment and Fkbp5 knockdown did not significantly influence the total insulin contents (Fig. S2C). This result further comfirmed that FKBP5 inhibition protects β-cell function from inflammation stress.

### FKBP5 inhibition activates Akt/Foxo1 signaling in β cells

**Fig. 4:**
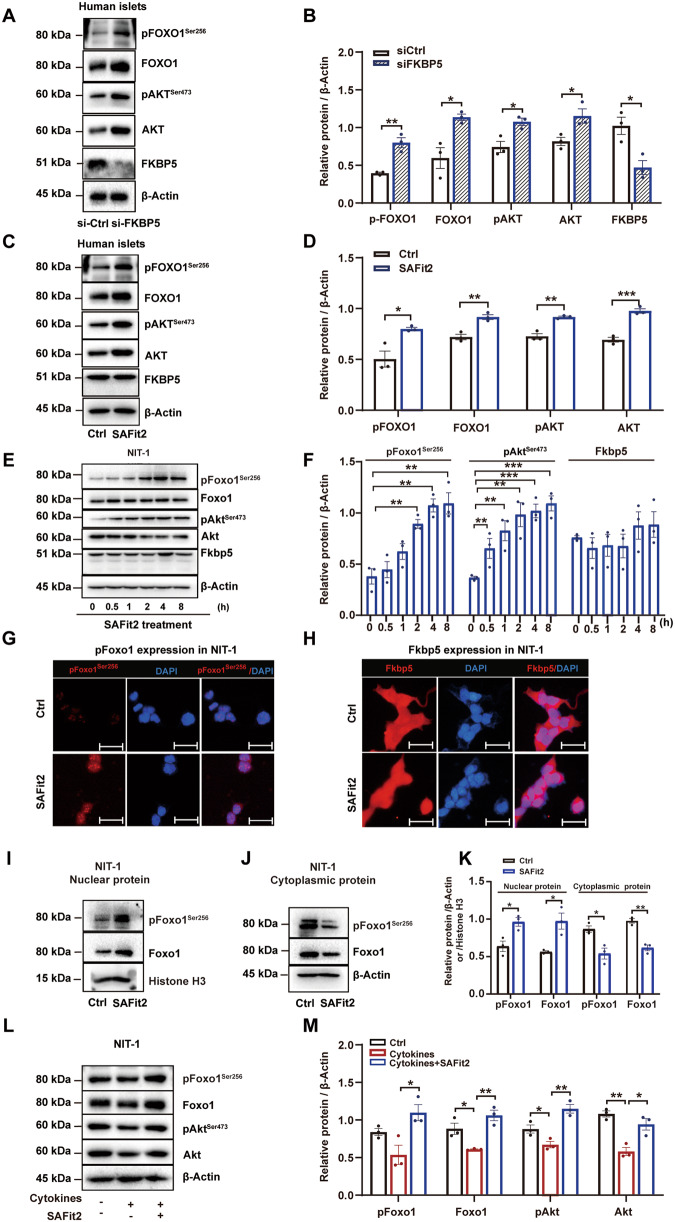
FKBP5 inhibition promotes FOXO1 phosphorylation. **A**, **B** Western blot analysis of FKBP5, p-FOXO1^Ser256^, FOXO1, p-AKT ^Ser473^, and AKT in primary human islets transfected with siFKBP5 or control siRNAs for 48 h (**A**). Relative proteins expression levels were quantified by Image J (**B**). **C**, **D** Western blot analysis of p-FOXO1^Ser256^, FOXO1, p-AKT^Ser473^, and AKT expressions in primary human islets treated with SAFit2 or control vehicle for 48 h (**C**). Relative proteins expression levels were quantified by Image J (**D**). **E**, **F** Western blot analysis of p-Foxo1^Ser256^, Foxo1, p-Akt^Ser473^, Akt and Fkbp5 in NIT-1 cells treated with SAFit2 (1 µM) for 0, 0.5, 1, 2, 4, 8 h (**E**). Relative proteins expression levels were quantified by Image J (**F**)**. G**, **H** Immunofluorescence staining with p-Foxo1 (red) (**G**) or Fkbp5 (red) (**H**), insulin (Ins, green) and DAPI (blue) in NIT-1 cells treated with SAFit2 or control vehicle for 24 h. Scale: 20 μm. **I**–**K** Western blot analysis nuclear (**I**) and cytoplasmic (**J**) protein of p-Foxo1^Ser256^ and Foxo1 in NIT-1 cells treated with SAFit2 or control vehicle. Relative proteins expression levels were quantified by Image J (**K**). **L**, **M** Western blot analysis of p-Foxo1^Ser256^, Foxo1, p-Akt^Ser473^, Akt, expression in NIT-1 cells treated with proinflammatory cytokines, or Cytokines + SAFit2 (**L**). Relative proteins expression levels were quantified by Image J (**M**). Experiments were repeated for 3 times. Student’s *t*-test. Mean ± SEM, *P < 0.05, **P < 0.01, ***P < 0.001.

FKBP5 is a multi-functional protein [17–19], yet SAFit2 specifically antagonizes its interaction with PHLPP and hence inhibits the downstream dephosphorylation of AKT at Serine 473^Ser473^, resulting in the accumulation AKT-Ser^473^ and the subsequent Serine 256 phosphorylation^Ser256^ of FOXO1, its classic downstream effector. FOXO1 is not only a critical transcription factor for insulin gene in β cells, but also plays a key role in β-cell survival under stresses, such as oxidative stress [20, 21] and hypoxia [22]. Here, using siRNAs we achieved a 54% knockdown of FKBP5 expression in primary human islets (Fig. 4A, B). As expected, FKBP5 knockdown significantly upregulated the phosphorylation level of AKT at Ser^473^ and FOXO1 at Ser^256^ (Fig. 4A, B). Similar results were also observed in SAFit2-treated human islets (Fig. 4C, D). We further confirmed the activation of AKT/FOXO1 signaling by a time-course study. NIT-1 cells were incubated with SAFit2 for 0, 0.5, 1, 2, 4, 8 h, and western blot assays showed that p-Akt^Set473^ began to increase at 0.5 h, and p-Foxo1^Ser256^ began to increase at 2 h (Fig. 4E, F), which lasted till 24 h after treatment (Fig. S3). Together, these results indicated that FKBP5 inhibition activated AKT/FOXO1 signaling.

Consistently, in inflammation treated NIT-1 cells, western blot results showed that the expression levels of phosphorylated Akt at Ser^473^ and Foxo1 at Ser^256^ were significantly decreased in response to proinflammatory cytokines treatment (Fig. 4L, M), suggesting that Akt/Foxo1 signaling is inhibited in inflammatory treated β cells. However, SAFit2 pretreatment remarkably restored their expressions (Fig. 4L, M), indicating that SAFit2 eliminated the inhibition effect of inflammation on Akt/Foxo1 signaling. This result hinted that Akt/Foxo1 signaling might be the key player in mediating the protective effect of FKBP5 inhibition on β cells facing inflammation.

### p-Foxo1^Ser256^ accumulates in nucleus of β cells upon FKBP5 inhibition

A critical difference in FOXO1 expression in β cells and other cell types is its subcellular localization. In other cells, such as cancer cells and hepatocytes, FOXO1 is primarily located in the nucleus at homeostatic state; yet it is constitutively located in the cytoplasm in homeostatic β cells and undergoes nucleus translocation in stressed β cells [20, 23]. To explore the localization of p-Foxo1^Ser256^ in β cells, cyto-immunofluorescence with p-Foxo1^Ser256^ was performed in NIT-1 cells. The result showed that in control NIT-1 cells p-Foxo1^Ser256^ immune-reactivity was weak and restricted to the nucleus but in SAFit2-treated cells p-Foxo1^Ser256^ immune-reactivity was significantly enhanced (Fig. 4G). In addition, cyto-immunofluorescence with FKBP5 demonstrated that the immune-reactivity and localization of FKBP5 had no obvious alteration in SAFit2-treated cells (Fig. 4H). Furthermore, the expression changes of p-Foxo1^Ser256^ and total Foxo1 in cytoplasm and nucleus, respectively, in NIT-1 cells treated with or without SAFit2 were determined by western blot using the cytoplasm and nucleus proteins extracts. Results showed that SAFit2 treatment significantly increased the amount of nuclear p-Foxo1^Ser256^ and Foxo1 (Fig. 4I, K), but decreased the level of cytoplasmic p-Foxo1^Ser256^ and Foxo1 (Fig. 4J, K). These results suggested a unique nuclear distribution of p-Foxo1^Ser256^ protein in β cells and its expression level in neuleus was further enhanced by FKBP5 inhibition.

### The protective effect of FKBP5 inhibition on β-cell function is Foxo1 dependent

**Fig. 5:**
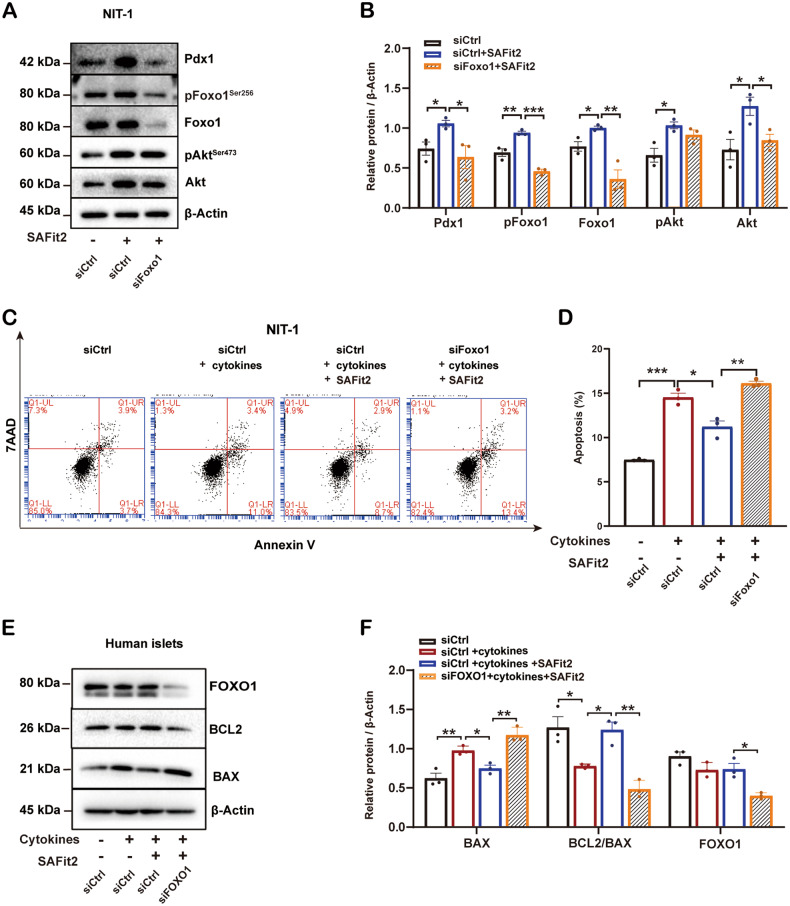
FOXO1 mediated the protective effect of FKBP5 inhibition on β cells. **A**, **B** Western blot analysis of Pdx1, p-Foxo1^Ser256^, Foxo1, p-Akt ^Ser473^ and Akt in NIT-1 cells transfected with siFoxo1 or control siRNAs, and then treated with SAFit2 (**A**). Relative proteins expression levels were quantified by Image J (**B**). **C**, **D** Annexin-V/7-AAD staining and flow cytometry analysis in NIT-1 cells transfected with siFoxo1 or control siRNAs and then treated with cytokines, or cytokines + SAFit2 (**C**). Quantification of PE^+^7AAD^+^/PE^+^7AAD^-^ cells (**D**). **E**, **F** Western blot analysis of FOXO1, BCL2, BAX in primary human islets transfected with siFOXO1 or control siRNAs and then treated with cytokines, or cytokines + SAFit2 (**E**). Relative proteins expression levels were quantified by Image J (**F**). Experiments were repeated for 3 times. Student’s *t*-test. Mean ± SEM, *P < 0.05, **P < 0.01, ***P < 0.001.

To investigate whether Foxo1 mediates the protective effects on β cells with SAFit2, siRNAs were used to silence the Foxo1 gene in NIT-1 cells. Western blot assay showed that the Foxo1 protein expression was significantly decreased by 67% in siFoxo1 cells (Fig. S4). We further treated the siCtrl cells and the siFoxo1 cells with SAFit2, and western blot results demonstrated that SAFit2 treatment in siCtrl cells enhanced the protein expression of Pdx1, a critical β-cell functional gene, but this effect was abolished in siFoxo1 cells (Fig. 5A), suggesting that Foxo1 knockdown abrogated the improvement of SAFit2 to β-cell function of siCtrl cells. In addition, in siCtrl cells, SAFit2 treatment upregulated the expression levels of phosphorylated Foxo1 at Ser^256^ and Akt at Ser^473^ (Fig. 5A, B); yet in siFoxo1 cells only p-Akt had a comparable expression level with that in siCtrl cells, suggesting that the knockdown of Foxo1 did not influence the activation of Akt by SAFit2 (Fig. 5A, B). Together, these results suggested that the protective effect of FKBP5 inhibition on β-cell function depends on the activation of Akt/Foxo1 signaling.

### Foxo1 mediates SAFit2 to reduce inflammation-induced β-cell apoptosis

To further investigate whether Foxo1 is indispensable in the regulation of β-cell survival mediated by SAFit2 under inflammatory stress, we evaluated the levels of apoptosis with siRNA knockdown of Foxo1 in NIT-1 cells by Annexin-V/7-AAD staining. We found a significant increase level of apoptotic rate under inflammatory stress in NIT-1 cells, and this trend was reversed in the presence of SAFit2. However, the silencing of Foxo1 abolished the protective function induced by SAFit2 under inflammatory stress (Fig. 5C, D). Furthermore, we verified this result in human islets. Western blot assays showed that inflammation cytokines treatment significantly upregulated BAX expression and downregulated BCL2 expression, as well as the ratio of BCL2 to BAX, indicating inflammation treatment impaired β-cell surviving ability. In the presence of SAFit2 treatment, the expression level of BAX and BCL2 were restored, confirming the protective effect of SAFit2 against inflammation insults in human islets. However, this protective function of SAFit2 was abolished after FOXO1 silencing (Fig. 5E, F). These results together demonstrated that Foxo1 plays an indispensable role in mediating the protection of SAFit2 from inflammatory stress in β cells.

### Reduced FKBP5 expression in islet β cells of human T2D patients and db/db mice

**Fig. 6:**
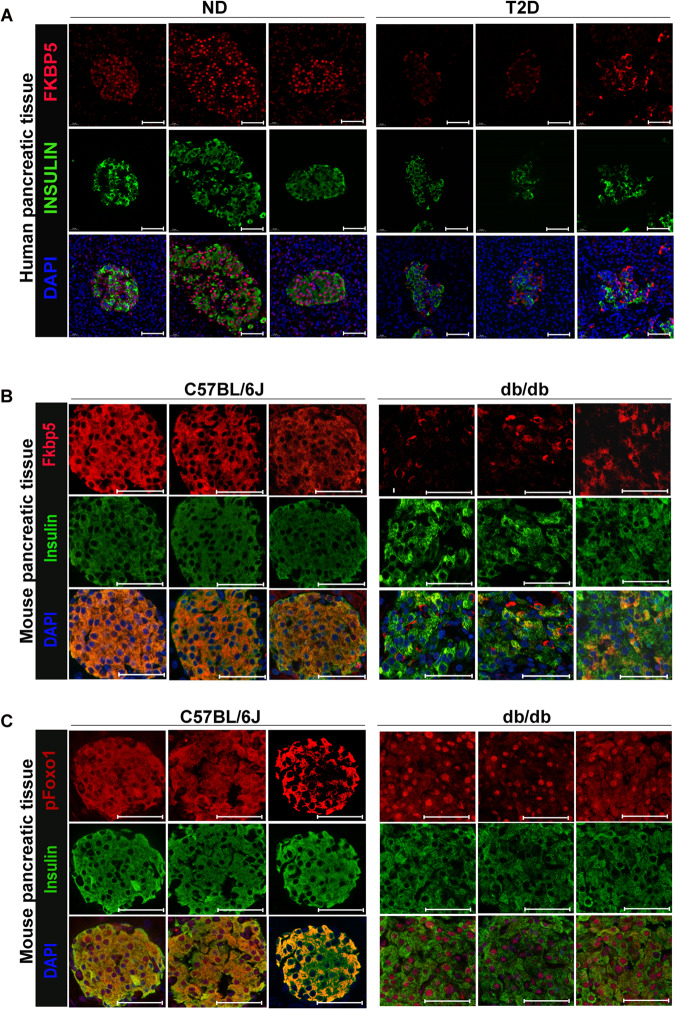
Reduced FKBP5 expression in islet β cells of human T2D patients and db/db mice. **A** Immunofluorescence staining with FKBP5 and insulin in human pancreatic tissues of ND and T2D subjects. *n* = 3. Red: FKBP5, Green: Insulin,.Blue: DAPI. Scale: 50 μm. **B** Immunofluorescence staining with Fkbp5 and insulin in mice pancreatic tissues of C57BL/6J and db/db mice. *n* = 3. Red: Fkbp5, Green: Insulin, Blue: DAPI. Scale: 50 μm. **C** Immunofluorescence staining with pFoxo1 and insulin in mice pancreatic tissues of C57BL/6J and db/db mice. *n* = 3. Red: pFoxo1, Green: Insulin, Blue: DAPI. Scale: 50 μm.

Although we have found that acute-inflammation treatment in vitro induced FKBP5 expression, it was unknown whether and how FKBP5 expression changes in T2D, where long-term and complex stresses exist. Here, using immunofluorescence staining with FKBP5 and insulin in human pancreatic tissues from non-diabetic or T2D individuals, we found that FKBP5 has a substantial expression in β cells in non-diabetics, but the expression intensity significantly decreased in T2D; in non-diabetics, there were few β cells without FKBP5 expression, but this phenomenon is more common in T2D islets (Fig. 6A). Interestingly, FKBP5 protein exhibited a nuclear localization in human pancrease, which is likely due to the warm and cold ischemia processes caused oxidative stress during pancreas procurement [24] To further validate the FKBP5 expression level in diabetic conditions, we examined the Fkbp5 in situ expression in the pancreas of a diabetic mice model, db/db mice. Consistently, the immunostaining results demonstrated that Fkbp5 expression was also markedly decreased in db/db mice compared to that in control mice (Fig. 6B). Moreover, immunofluorescence staining with p-Foxo1^Ser256^ revealed that nuclear p-Foxo1^Ser256^ significantly increased in db/db mice pancreases (Fig. 6C), in accordance with the corresponding low expression of Fkbp5. These results indicated that FKBP5 expression in β cells in T2D is downregulated in the long run.

## Discussion

Our results demonstrated that FKBP5 depletion by siRNAs promoted β-cell survival under inflammation stress, and hence improved glucose-stimulated insulin secretion in ex vivo-cultured human and mice islets, accompanied with increased expression of β-cell key transcription factors. SAFit2, a pharmacological antagonism of FKBP5, recapitulated the effects of FKBP5 deletion. We further identified that AKT /FOXO1 signaling mediates the protective effect of FKBP5 inhibition and FKBP5 is a key regulator of FOXO1 functioning in β cells.

A striking finding of this study is the discovery of FKBP5 expression in both human islets and mice islets, especially in β cells. Pioneering studies have reported the abundant expression of FKBP5 in liver and adipose tissue [25]. However, its expression in islets have not been reported. Western blot assays even showed a negative expression of FKBP5 in pancreas tissue, which might be due to the low occupation of islets in pancreas at physiological condition. Here, we discovered that FKBP5 is also expressed in pancreatic islets, especially in β cells, at both mRNA and protein level, evidenced by qPCR, WB, and immunofluorescence assays. Using siRNAs to deplete FKBP5 expression and pharmacologically inhibiting the FKBP5 signaling, we demonstrated that FKBP5 inhibition improved β-cell functional gene expressions, glucose-stimulated insulin secretion in both human and mice islets, and promoted β-cell survival under inflammation stress. These results established the basic function of FKBP5 in β cells.

We found that FKBP5 expression was induced by inflammation stimulus, an inevitable stress for β cells during T2D development and progression. Further studies revealed that FKBP5 participates the regulation of β-cell survival under inflammation insults, and this protective effect of FKBP5 inhibition might be fulfilled by the activation of autophagy. FKBP5 has been reported as a regulator of autophagy in other tissue. In malignant melanoma, it has been reported that FKBP5 took part in the apoptosis resistance and promoted autophagy response to ionizing radiation [26]. In animal models of Huntington disease, reduction of FKBP5 expression increased LC3-II levels and autophagic flux [27]. Recently, Nils C. Gassen and his colleges demonstrated that FKBP5-regulated autophagy play a crucial role in mediobasal hypothalamus (MBH) under metabolic stress [28, 29]. However, whether FKBP5 regulates autophagy in β cells was previously unknown. Here, we firstly demonstrated that FKBP5 inhibition by siRNAs or inhibitor SAFit2 activates the autophagy flux in inflammation cytokines treated β cells, which may facilitate β-cell survival under inflammation stress. In addition, inflammation is also a common stress for islet isolation [30]. In the ex vivo-cultured human islets and mice islets, we found that FKBP5 inhibition improved β-cell function, evidenced by the increased expression of β-cell functional genes NKX6.1 and PDX1 and by the enhanced glucose-stimulated insulin secretion, confirming the protective effect of FKBP5 in β cells. This finding indicated that FKBP5 inhibitor can also serve as an additive in the islet culture before clinical islet transplantation.

We identified that FKBP5 is a key upstream regulator for FOXO1 in β cells. FOXO1 is a key transcription factor in β cells, regulating β-cell differentiation [31, 32], maintaining the mature state of β cells [33], and responsive to multiple metabolic stresses in diabetic milieus [34]. Although the importance of FOXO1 in β cells has been extensively studied, to the best of our knowledge, whether FKBP5 is an upstream regulator for FOXO1 has never been documented in β cells. FKBP5 is a key regulator of AKT activity and the downstream activation of nuclear receptors and other regulatory factors [35, 36]; meanwhile, FOXO1 is also a downstream effector of AKT [37]. In this study, we found that FKBP5 depletion by siRNAs promotes FOXO1 phosphorylation at Ser^256^ in human islets, and so did its inhibitor SAFit2. Previous studies have reported that FOXO1 protein can be modified by phosphorylation or acetylation in β cells, which precisely regulates FOXO1’s transcriptional activity, subcellular localization, and turnover [34, 38]. The phosphorylation of Foxo1 at Ser^256^ promotes its nucleus exclusion [21, 39, 40], but the acetylation of Foxo1 induced by metablic stress overrides phosphorylation-dependent nuclear exclusion and causes nuclear retention [20]. The net outcome of acetylation-dependent nuclear retention of Foxo1 is to increase expression of NeuroD and MafA, prevent β-cell replication under conditions that could result in apoptosis [20]. Here in this study, we observed the accumulation of nuclear p-Foxo1^Ser256^ in NIT-1 cells with FKBP5 inhibition by SAFit2 (Fig. 4) and β cells in db/db mice pancreases which also have reduced FKBP5 expression (Fig. 6), which is in support to the concept that Foxo1 exists in multiple nuclear forms with distinct activities depending on the balance of acetylation and phosphorylation [40]. Meanwhile, it is intriguing to thoroughly explore the mechanism for FKBP5-regulated nuclear accumulation of p-Foxo1, which will be one of our future endeavors.

Meanwhile, we displayed that AKT/FOXO1 pathway is the key signaling to mediate the protective effect of FKBP5 inhibition in β cells. In NIT-1 cells, silencing of FOXO1 by siRNAs abolished the upregulation of β-cell functional gene PDX1 by SAFit2. In inflammation-induced β-cell apoptosis, SAFit2 treatment improved β-cell survival, but this improvement disappeared when Foxo1 was silenced by siRNAs. This suggested that the execution of FKBP5 function in β cells depends on FOXO1 signaling. In addition, earlier studies, including ours, have reported FOXO1’s crucial role in promoting β-cell survival via activating autophagy in both human and rodent β cells under different stresses, such as hypoxia and lipotoxicity [22, 41]. In this study, we further expanded our understanding on the regulatory role of FOXO1 in β cells survival against inflammation factors. We found that inflammation-induced β cells apoptosis with reduced Foxo1 phosphorylation at Ser^256^, but SAFit2 treatment alleviated inflammation-induced β cells apoptosis by promoting Foxo1 phosphorylation at Ser^256^. These results together indicated that FKBP5 inhibition promotes FOXO1 phosphorylation at Ser^256^ and its nucleus translocation under inflammatory stress, and hence increasing the transcription of key genes that can promotes β-cells survival.

Taking advantages of the pancreatic tissues from organ donors, we explored the expression of FKBP5 in human β cells both in non-diabetic and type 2 diabetic pancreas. Immunofluorescence staining of FKBP5 revealed a downregulation of the fluorescence intensity and an increase of FKBP5 low expression or even negative β cells, suggesting a decrease of FKBP5 expression in the β cells of T2D patients. This expression change of FKBP5 is in accordance with previously documented FOXO1 expression in T2D. A couple of studies, including ours, have reported the increase of β cells with loss of cytoplasmic expression of FOXO1, which has been a marker for β-cell dedifferentiation [42–44]. Since we have proved the regulatory role of FKBP5 on FOXO1 phosphorylation at Ser^256^, which mediates the subcellular localization of the protein, this result suggested that the decrease of FKBP5 expression in β cells in T2D may be responsible for the increased nucleus translocation of FOXO1 and the subsequent loss of cytoplasmic expression of FOXO1. Interestingly, although acute stresses treatment in vitro, such as cytokines-mimicked inflammation, high-glucose-mimicked glucotoxicity, and palmitic acid-mimicked lipotoxicity, stimulated the transcription of FKBP5, its expression finally reduced in T2D in the long run. The stresses faced by β cells in T2D are complex [45], and mechanisms for the reduction of FKBP5 protein in β cells in vivo in T2D patients worth further investigation.

In summary, this study discovered the expression of FKBP5 in β cells and clarified its function. FKBP5 inhibition protects β-cell survival in acute-inflammation stress and hence improving β-cell function. FKBP5 is an important regulator for FOXO1 andat the same time, FOXO1 is a critical mediator for FKBP5 functioning in β cells. Cross-sectional study in human pancreas revealed a reduced expression of FKBP5 in β cells of T2D patients, indicating a FKBP5-inhibition-mediated pro-survival mechanism against the complex stresses in type 2 diabetic milieus.

## Materials and methods

### Human pancreas tissue sections and human islets

Human pancreas tissue sections (3 µm, paraffin embedded) with T2D or non-diabetes (ND) were obtained from the Human Islet Resource Center (HIRC, China), Tianjin First Central Hospital, People of the Republic of China, with informed research consent. Human islets were prepared in HIRC, China, from the pancreases from organ donors with informed research consent. Human islets were isolated by Collagenase NB1 (SERVA, Heidelberg, Germany) and Neutral Protease NB (SERVA, Heidelberg, Germany) digestion and continuous density purification, according to our earlier documents [22, 42]. High purity islets (>80%) were collected and cultured in CMRL-1066 medium (Corning, Manassas, VA, USA), supplemented with 10% Human Serum Albumin (Baxter, Vienna, Austria), 100 U/mL penicillin, and 100 mg/mL streptomycin, at 37 °C in 5% CO_2_. Organ donor information including age, gender, diabetes status, hemoglobin A1c (HbA1c), and body mass index (BMI) was displayed in Table 1. All study protocols were approved by the Medical Ethical Committee of Tianjin First Central Hospital (2016N077KY).

**Table 1:** Organ donor characteristics.

Group	Donor ID	Age (years)	Gender	BMI (kg/m^2^)	HbA1c (%)
ND	HP033	59	M	30.42	5.7
ND	HP046	52	M	20.76	5.1
ND	H095	55	F	23.88	5.6
ND	H134	29	M	21.97	5.3
ND	H156	-	F	26.29	-
ND	H159	28	M	24.22	5.3
ND	H164	42	M	24.22	-
ND	H171	48	M	22.34	-
ND	H185	23	M	26.23	-
ND	H186	39	M	20.52	-
ND	H197	36	M	20.05	-
ND	H201	52	M	24.49	-
T2D	HP055	64	F	23.43	8.9
T2D	HP099	51	M	22.49	11.9
T2D	HP105	57	F	24.97	5.8

*ND* non-diabetes, *T2D* type 2 diabetes, *M* male, *F* female, *BMI* body mass index, *HbA1c* Hemoglobin A1c.

### Mice pancreas tissue sections and mice islets

Male ICR (8 to 10 weeks), C57BL/6J (11 weeks) and db/db (11 weeks) mice were purchased from Beijing Huafukang Biosciences (Beijing, China). All mice were fed standard chow diet and maintained on a 12 h light-dark cycle (lights on at 7:00 AM). Mice pancreas tissue sections (3 µm, paraffin embedded) from C57BL/6J and db/db mice. Islets were isolated from ICR mice, and the pancreatic tissue was perfused with collagenase P (0.5 mg/mL, Roche, Basel, Switzerland) and incubated on ice for 30 min, followed by digestion at 37°C for 11 min and purification by density gradient (Histopaque 1077, Sigma-Aldrich, St Louis, MO, USA). Isolated islets were either used immediately or cultured in RPMI 1640, supplemented with 10% FBS, 100 U/mL penicillin, and 100 mg/mL streptomycin, at 37°C in 5% CO_2_.

### Cell culture

Mice pancreatic β-cell lines βTC-6 (ATCC CRL-11506TM) and NIT-1 (ATCC CRL-2055TM) were used in this study. βTC-6 was cultured in DMEM medium, supplemented with 10% FBS, and NIT-1 cells were cultured in DMEM/F-12 medium, supplemented with 10% FBS at 37°C in 5% CO_2_. Both media were supplied with 100 U/mL penicillin and 100 μg/mL streptomycin.

### Drug treatment

SAFit2 (Aobious, AOB6548) was dissolved in dimethyl sulfoxide (DMSO, MilliporeSigma, D2650) to a final concentration of 1 mM. Cells were treated with DMSO or SAFit2 (1 μM) for 0, 0.5, 1, 2, 4, 8 h, 16 h, 24 h, and 48 h. When the cell confluency reached 80%, SAFit2 was added to fresh culture medium as part of the medium change.

### siRNA knockdown of FKBP5/FOXO1

Human islets, mice islets or NIT-1 cells were cultured as described above. Small interfering RNA (siRNA) against FKBP5 and FOXO1 for human and Fkbp5/Foxo1 for mice were purchased from GenePharma (Shanghai, China). Lipofectamine 3000 (Invitrogen, Carlsbad, CA, USA) was used to transfect siRNA, according the manufacturer instructions. mRNA and protein expression analyses were performed after transfection for 48 h.

### RNA isolation and qRT-PCR

Total RNA was extracted from cultured cells using Trizol Reagent (Invitrogen, Carlsbad, CA, USA), according to the manufacturer’s instructions. Total RNA was reverse transcribed to cDNA using reverse transcriptase (RT) reaction kit (Takara, Kohoku-Cho, Kusatsu, Japan). qRT-PCR was performed using FastStart Essential DNA Green Master on a LightCycler96 machine (Roche, Basel, Switzerland). The relative expression of mRNA to internal control (Arppo RNA) was calculated using the 2^-ΔΔCt^ method. The primers used in this study, human genes included: (1) *36B4-*F: 5′-AGGCGTCCTCGTGGAAGTGA-3′, 36B4-R: 5′-GCGGATCTGCTGCATCTGCT-3′ ; (2) *FKBP5*-F: 5′-CTCCCTAAAATTCCCTCGAATGC-3′, *FKBP5*-R: 5′-CCCTCTCCTTTCCGTTTGGTT-3′; (3) *NKX6.1*-F:5′-GGGCTCGTTTGGCCTATTCGTT-3′, *NKX6.1*-R: 5′-CCACTTGGTCCGGCGGTTCT-3′; (4) *MAFA-*F: 5′-GCTCTGGAGTTGGCACTTCT-3′, *MAFA-*R: 5′-CTTCAGCAAGGAGGAGGTCA-3′; (5) *INS-*F: 5′-GCAGCCTTTGTGAACCAACAC-3′, *INS-*R: 5′-CCCCGCACACTAGGTAGAGA-3′. Mice primer used included: (1) Arppo-F: 5′-GTGACGTTGACATCCGTAAAGA-3′, Arppo-R: 5′-GCCGGACTCATCGTACTCC-3′; (2) *Fkbp5*-F: 5′-TTTGAAGATTCAGGCGTTATCCG-3′, *Fkbp*5-R: 5′-GGTGGACTTTTACCGTTGCTC-3′; (3) *Nkx6.1*-F: 5′-CTGCACAGTATGGCCGAGATG-3′, *Nkx6.1*-R: 5′-CCGGGTTATGTGAGCCCAA-3′; (4) *Mafa*-F: 5′-AGGAGGAGGTCATCCGACTG-3′, *Mafa*-R: 5′-CTTCTCGCTCTCCAGAATGTG-3′; (5) *Ins1*-F: 5′-GAAGTGGAGGACCCACAA GTG-3′, *Ins1*-R: 5′-ATCCACAATGCCACGCTTCT-3′; (6) *Ins2*-F:5′-GAAGTGGAGGACCCACAAGTG-3′, *Ins2*-R:5′-GATCTACAATGCCACGCTTCTG -3′.

### Western blot

Total proteins were harvested from culture dishes and lysed in RIPA buffer (Thermo Fisher Scientific, 89900, Waltham, MA, USA) supplemented with protease inhibitors and phosphatase inhibitors (Invitrogen, Carlsbad, CA, USA). Nuclear and cytoplasmic proteins were harvested from culture dishes and extracted by Nuclear Extraction Kit (Solarbio, SN0020, Beijing, China). Protein concentrations were determined using a BCA Protein Assay Kit (Solarbio, Beijing, China), and 10 µg protein was separated by SDS-PAGE for PVDF membrane blotting. The blotted membranes were blocked with 5% skim milk for 60 min at room temperature and incubated with primary antibodies FKBP51 (1:1000, ABclonal, A3863, Woburn, MA, USA), p-FOXO1 (1:1000, NB100-81927, Novus Biological, Cambridge, UK), FOXO1 (1:1000, CST-2880, Cell signaling technology, Danvers, MA, USA), p-AKT(1:1000, CST-4060S, Cell signaling technology, Danvers, MA, USA), AKT(1:1000, CST-9272S, Cell signaling technology, Danvers, MA, USA), PDX1(1:1000, CST-9679S, Cell signaling technology, Danvers, MA, USA), Sqstm1/p62 (1:1000, ab56416, Abcam, Cambridge, MA, USA), LC3I/II (1:1000, CST-12741S, Cell signaling technology, Danvers, MA, USA), BAX(1:1000, ab32503, Abcam, Cambridge, MA, USA), BCL2(1:1000, ab59348, Cambridge, MA, USA), β-actin (1:1000, CST-3700, Cell signaling technology, Danvers, MA, USA), Histone cluster 1, H3a (1:1000, 17168-1-AP, Proteintech, Rosemont, IL 60018, USA) at 4°C overnight. The immunoblots were visualized by enhanced chemiluminescence using horseradish peroxidase-conjugated IgG secondary antibodies and then quantified using ImageJ. Data from three independent experiments were presented following the corresponding blotting images in the figures.

### Glucose-stimulated insulin secretion (GSIS) assay

Ten islets were pretreated in 1 mL of 1.67 mM low-glucose Krebs-Ringer bicarbonate buffer (KRB; supplemented with 0.5% BSA, pH 7.4) for 1 h in a 12-well plate, followed by sequential treatment with 1 mL low-glucose KRB solution (1.67 mM) for 1 h and high-glucose KRB solution (16.7 mM) for 1 h. The media with low- and high-glucose levels were collected separately, and measured using an ELISA kit (Mercodia, Uppsala, Sweden). Ten islets were lysed by sonication with 1 mL RIPA buffer (Thermo Fisher Scientific, 89900, Waltham, MA, USA) on ice. Islet intracellular insulin and total protein concentration were measured using ELISA and a BCA protein assay kit (Solarbio, Beijing, China). Insulin secretion of islets was expressed as the glucose-stimulated index (GSI; insulin secretion at high-glucose/insulin secretion at low glucose). Insulin contents in low/high-glucose and intracelluar insulin contents were presented after being normalized by the total protein.

### Immunohistochemistry

Human or mice pancreas tissue were fixed in 4% paraformaldehyde, embedded with paraffin, and sectioned (3 mm). After deparaffinization, sections were treated with EDTA antigen retrieval solution (Solarbio, Beijing, China) in a microwave oven, washed, permeabilized, and blocked. Immunohistochemical staining was performed using FKBP5 (1:300, ABclonal, OH, USA) and p-Foxo1 (1:300, Novus Biological, Cambridge, UK), then incubation with TRITC AffiniPure Goat Anti-Rabbit IgG H&L (1:200, Jackson Immunoresearch Laboratories and Molecular Probes, West Grove, PA, USA) secondary antibodies. Counterstaining was performed with DAPI (Vector, Burlingame, CA, USA). We used Pannoramic MIDI and Pannoramic Viewer (3DHistech, Budapest, Hungary) to scan stained slides and capture images. The Image Pro-Plus software (Media Cybernetics, Silver Spring, Maryland) was used to quantified in a blinded fashion.

For immunostaining in cultured β cells, cells were washed three times in PBS, and the fixed in 4% PFA for 20 min, incubated 15 min in 0.05% Triton X-100, then washed three times in PBS and incubated with rabbit anti-Fkbp5 (1:300, ABclonal, OH, USA) and rabbit anti-p-Foxo1 (1:300, Novus Biological, Cambridge, UK) at 4 °C overnight. After washing, cells were incubated with Goat Anti-Rabbit IgG secondary antibodies (1:200, Jackson Immunoresearch Laboratories and Molecular Probes, West Grove, PA, USA) secondary antibodies and then counterstained with DAPI. Images were captured under fluorescent microscope.

### Apoptosis and autophagy analysis

The Annexin-V-PE/7-AAD apoptosis detection kit (BD Biosciences, Franklin lakes, NJ, USA) was used to assess the cell apoptosis level. Following the manufacturer’s, cells stained with PE conjugated Annexin-V and 7-Amino-Actinomycin D (7-AAD) in dark environment with room temperature for 15 min, and then underwent flow cytometry analysis (FACScan, BD Biosciences, Franklin lakes, NJ, USA). The early staged apoptotic cells (Annexin-V-PE^+^7-AAD^-^) and late staged apoptotic cells (Annexin-V-PE^+^7-AAD^+^) were together assumed as apoptotic cells.

Cell autophagy was detected using Cyto-ID Autophagy Detection Kit (Enzo Life Sciences, NY, USA), and propidium iodide (PI) staining was also used to exclude the apoptotic cells. Chloroquine (CQ) was used to block the autophagosome degradation, and the concentration of CQ was selected according to the previous study [22, 46]. Autophagy level was evaluated by flow cytometry, as described previously [47, 48].

### Statistics

Figure drawing and data processing were performed using GraphPad Prism v9.0 (GraphPad Software, 218 La Jolla, CA, USA). Student *t*-test was used for analyzing the group differences. Data are shown as mean ± SEM. *P* < 0.05 was considered statistically significant.

## Supplementary information

Reproducibility checklist

Supplementary Figure

Supplementary Material

## Data Availability

All data are available in the main text or the supplementary materials.
