# Supplementary material for: The inhibition of FKBP5 protects β-cell survival under inflammation stress via AKT/FOXO1 signaling
Source: Cell Death Discov. 2023 Jul 14;9:247. doi: 10.1038/s41420-023-01506-x (PMC10349081; doi:10.1038/s41420-023-01506-x)

Supplementary Figures

Figure S1. FKBP5 inhibition improved β cell function in primary human and mouse islets. A mRNA expression of *Fkbp5*, *Nkx6.1*, *Mafa*, *Ins1* and *Ins2* in primary mouse islets transfected with siFkbp5 or control siRNAs for 48 h. Experiments were repeated for 3 times. **B** GSI in primary mouse islets transfected with siFkbp5 or control siRNAs for 48 h. Data were generated from three repeated experiments. **C** mRNA expression of *FKBP5*, *NKX6.1*, *MAFA* and *INS* in primary human islets transfected with siFKBP5 or control siRNAs for 48 h. Experiments were repeated for 3 times. **D** GSI in primary human islets transfected with siFKBP5 or control siRNAs for 48 h. Data were generated from three repeated experiments. Student's *t*-test. Mean ± SEM, *$P < 0.05$, **$P < 0.01$, ***$P < 0.001$.

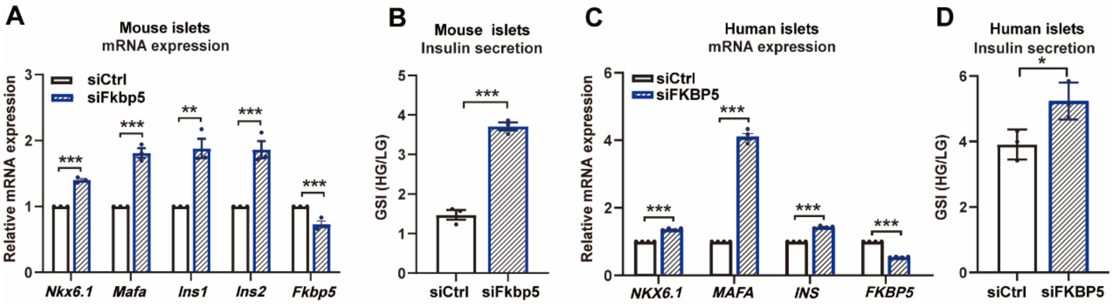

Figure S2. Fkbp5 inhibition improved β cell function in primary mouse islets treated with inflammatory cytokines. A-C GSI (A), insulin secretion (B), and intracellular insulin content (C) in primary mouse islets transfected with siFkbp5 or sictrl, and then treated with proinflammatory cytokines. Data were generated from three repeated experiments. Student's *t*-test. Mean ± SEM, *$P<0.05$, **$P<0.01$.

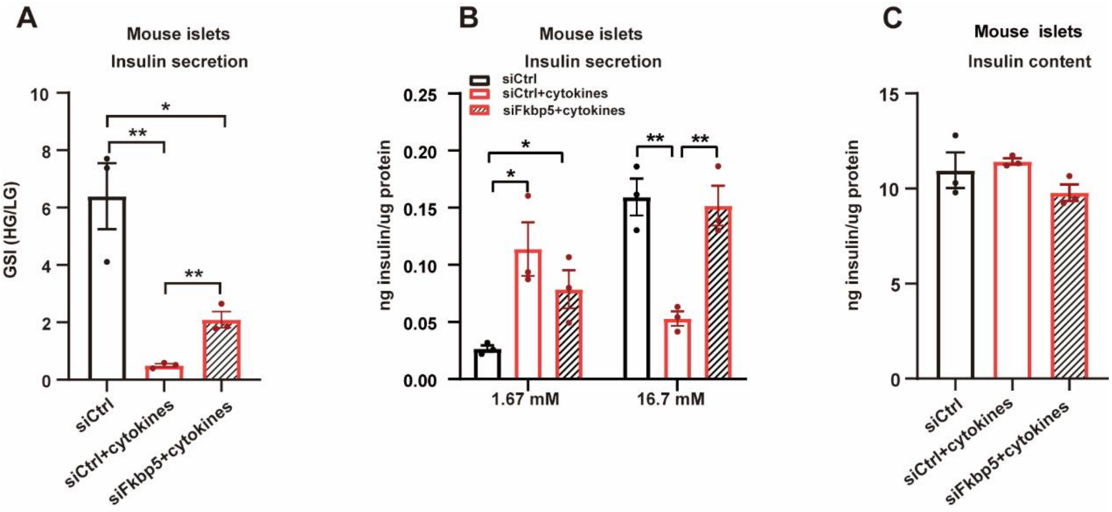

Figure S3. Fkbp5 inhibition activates Akt/Foxo1 signaling in NIT-1 cells. A Western blot analysis of p-Foxo1^{Ser256}, p-Akt^{Set473} and Fkbp5 in NIT-1 cells treated with SAFit2 (1 μM) for 0, 8, 16, 24, 48 h. **B** Relative protein expression levels were quantified by Image J. Experiments were repeated for 3 times. Student's *t*-test. Mean ± SEM, **P*＜0.05, ***P*＜0.01.

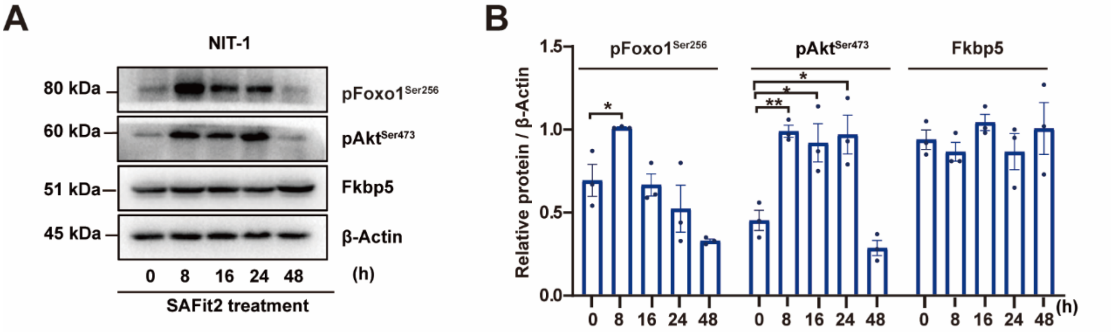

Figure S4. Foxo1 expression in NIT-1 cells transfected with siFoxo1 or control siRNAs. A Western blot analysis of Foxo1 in NIT-1 cells transfected with siFoxo1 or sictrl for 48 h. **B** Protein expression level of Foxo1 was quantified by Image J. Experiments were repeated for 3 times. Student's *t*-test. Mean ± SEM, $^{**}P < 0.01$.

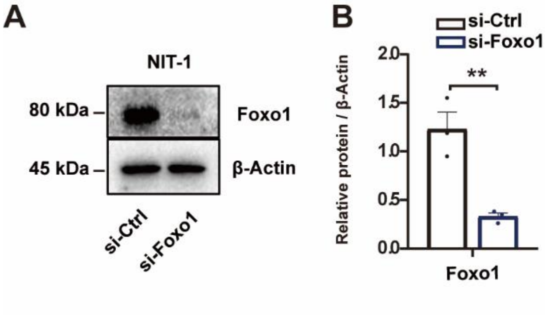

Supplement: Supplementary file 2 — Supplementary Figure [file 41420_2023_1506_MOESM2_ESM.pdf]
