# Supplementary material for: The inhibition of FKBP5 protects β-cell survival under inflammation stress via AKT/FOXO1 signaling
Source: Cell Death Discov. 2023 Jul 14;9:247. doi: 10.1038/s41420-023-01506-x (PMC10349081; doi:10.1038/s41420-023-01506-x)
Supplement: Supplementary file 3 — Supplementary Material [file 41420_2023_1506_MOESM3_ESM.pdf]

Fig1 D

Exp.1

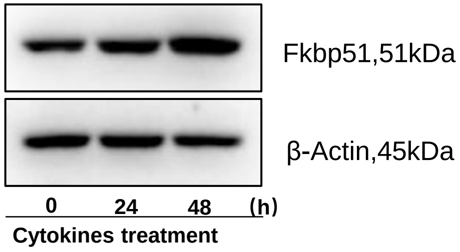

Gels: 12%, 15 well combs
Fkbp51: WX904284
β-Actin: CST3700

Exp.2

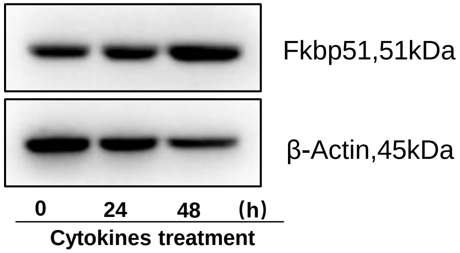

Gels: 12%, 15 well combs
Fkbp51: WX904284
β-Actin: CST3700

Exp.3

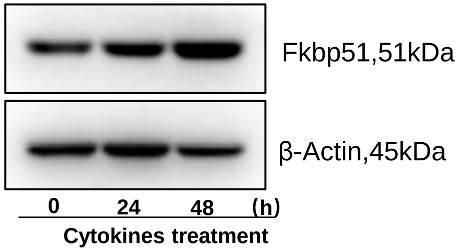

Gels: 12%, 15 well combs
Fkbp51: WX904284
β-Actin: CST3700

Fig2 D

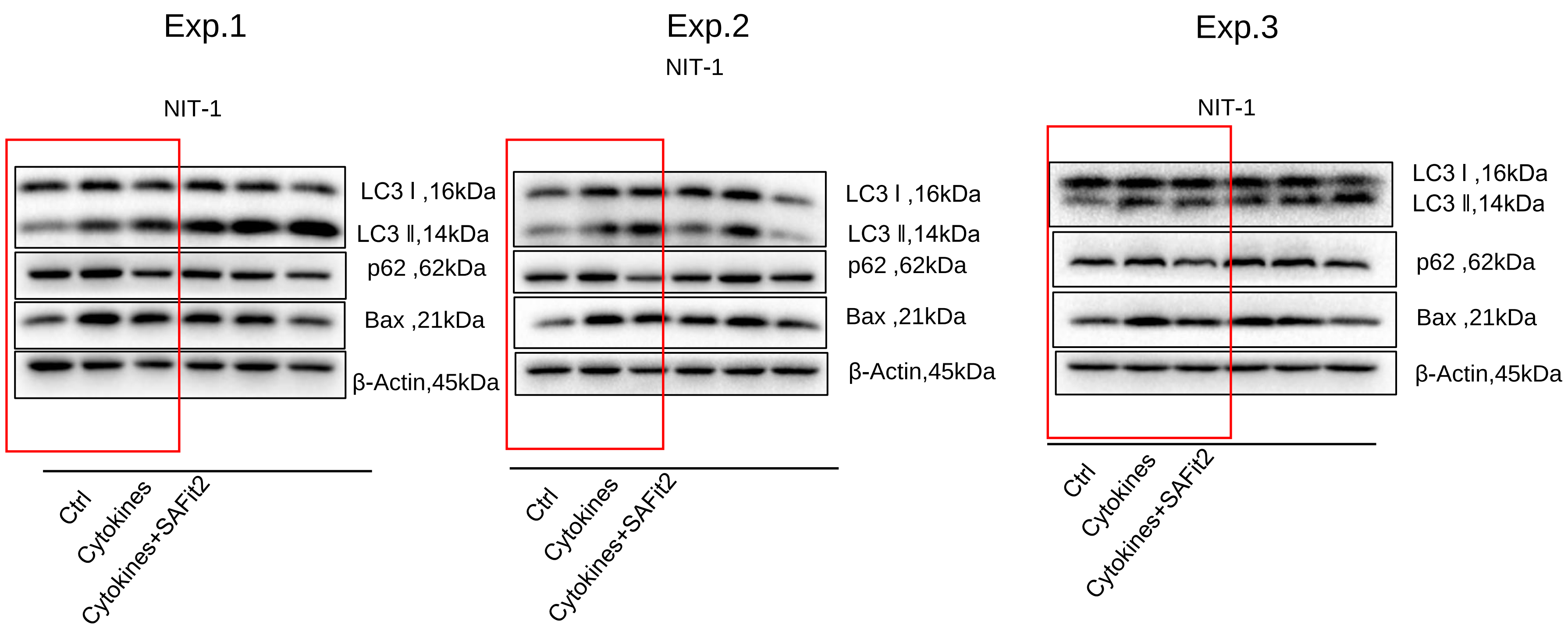

Gels: 15%, 15 well combs
p62: AB56416
Bax: ab32503
LC3I/II: CST12741S
β-Actin: CST3700

Gels: 15%, 15 well combs
p62: AB56416
Bax: ab32503
LC3I/II: CST12741S
β-Actin: CST3700

Gels: 15%, 15 well combs
p62: AB56416
Bax: ab32503
LC3I/II: CST12741S
β-Actin: CST3700

Fig4A

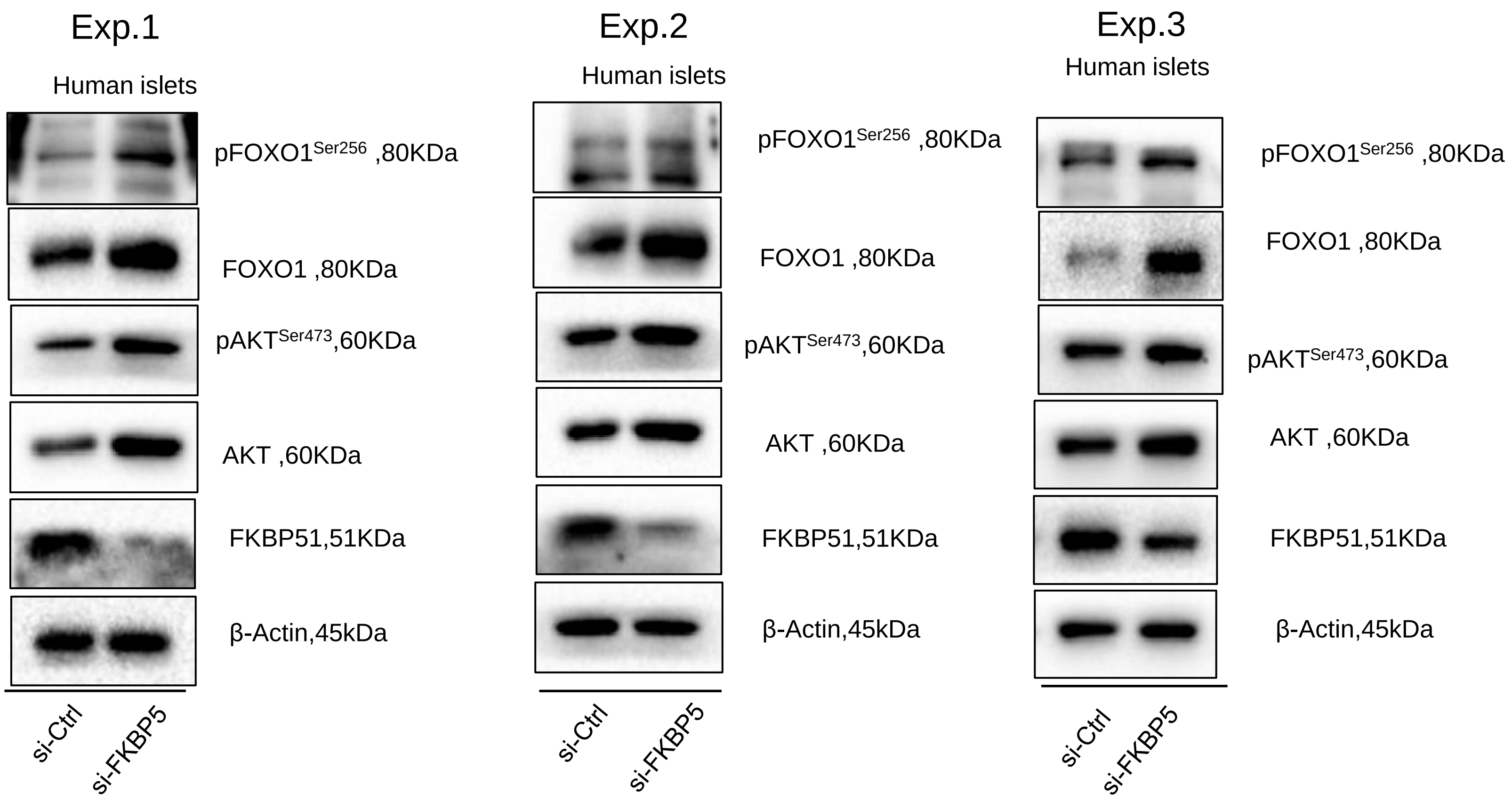

Gels: 10%, 15 well combs
pFOXO1: NB100-8192
FOXO1: CST-2880
pAKT: CST-4060S
AKT: CST-9272S
FKBP51: WX904284
β-Actin: CST3700

Gels: 10%, 15 well combs
pFOXO1: NB100-8192
FOXO1: CST-2880
pAKT: CST-4060S
AKT: CST-9272S
FKBP51: WX904284
β-Actin: CST3700

Gels: 10%, 15 well combs
pFOXO1: NB100-8192
FOXO1: CST-2880
pAKT: CST-4060S
AKT: CST-9272S
FKBP51: WX904284
β-Actin: CST3700

Fig4 C

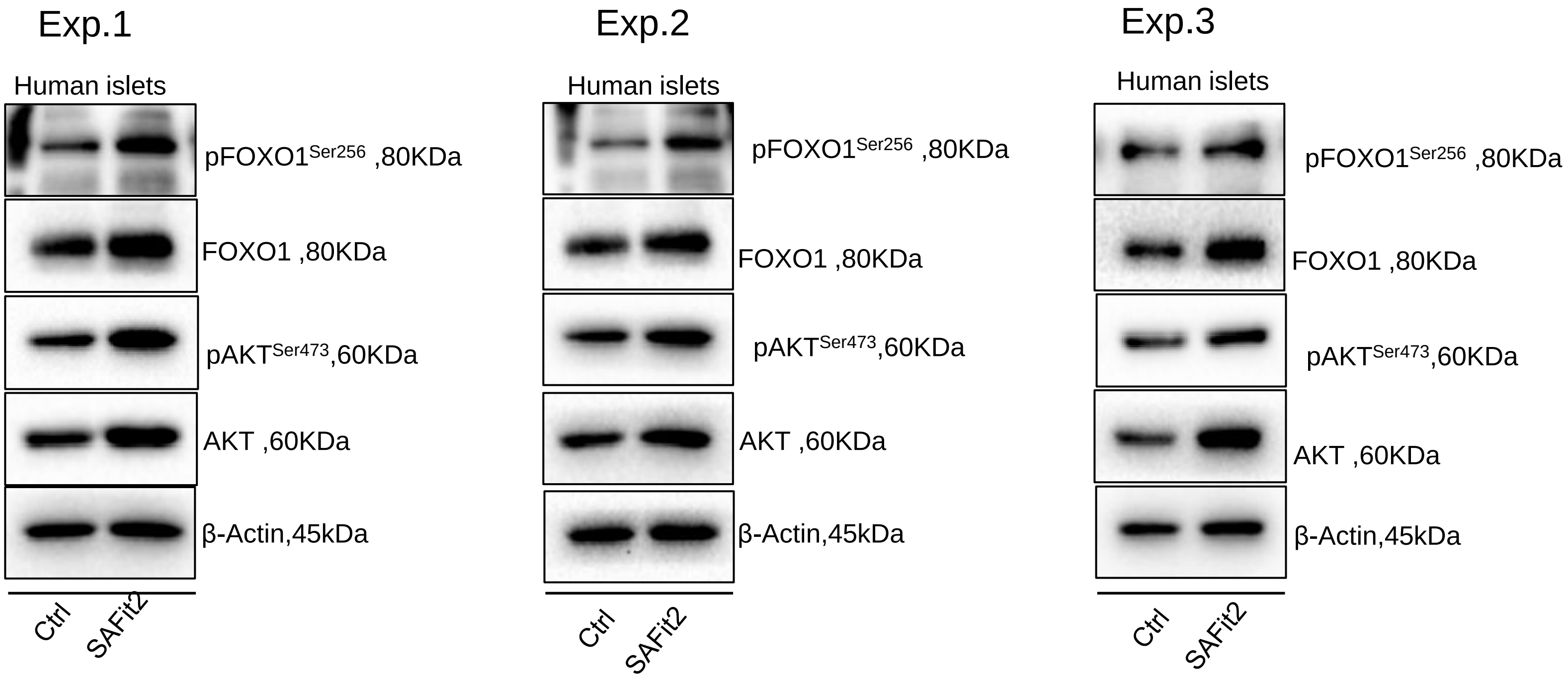

Gels: 10%, 15 well combs
P-FOXO1: NB100-8192
FOXO1: CST-2880
P-AKT: CST-4060S
AKT: CST-9272S
FKBP51: WX904284
β-Actin: CST3700

Gels: 10%, 15 well combs
P-FOXO1: NB100-8192
FOXO1: CST-2880
P-AKT: CST-4060S
AKT: CST-9272S
FKBP51: WX904284
β-Actin: CST3700

Gels: 10%, 15 well combs
P-FOXO1: NB100-8192
FOXO1: CST-2880
P-AKT: CST-4060S
AKT: CST-9272S
FKBP51: WX904284
β-Actin: CST3700

Fig4E

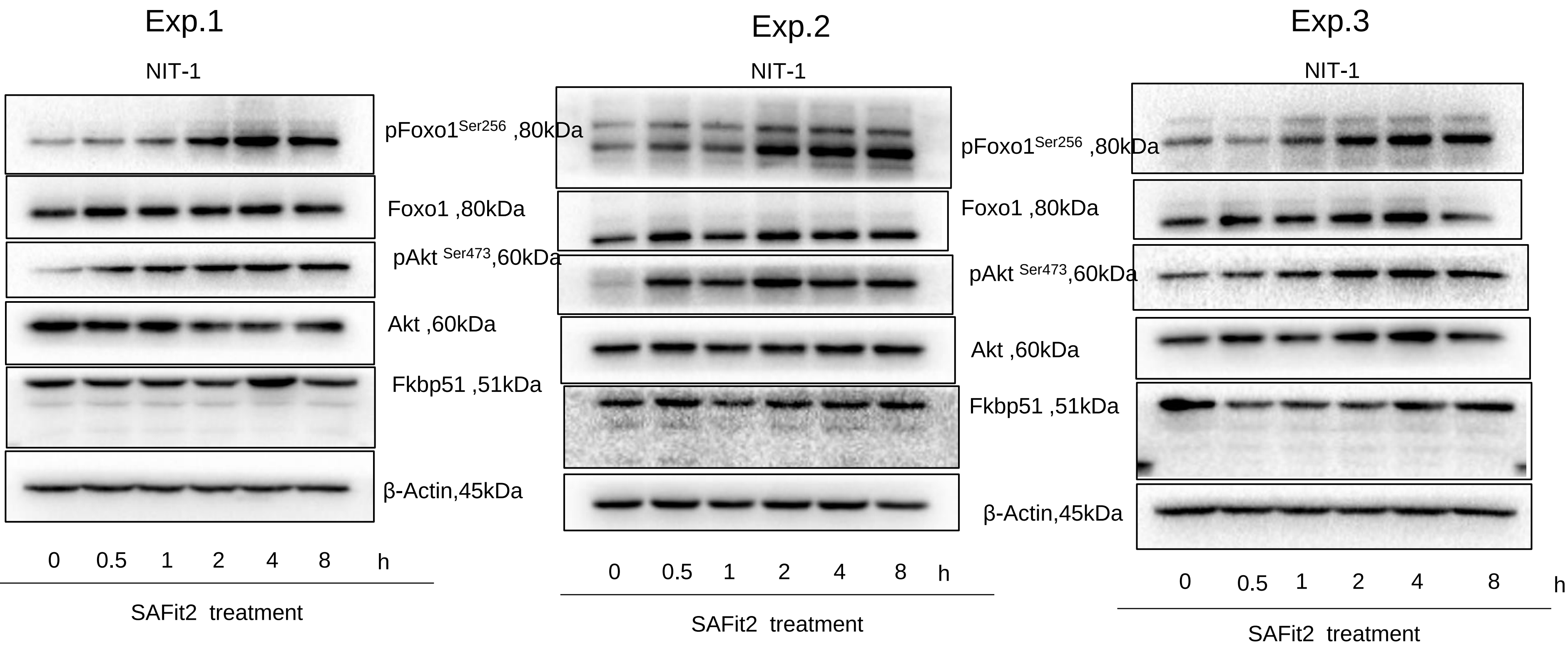

Gels: 12%, 15 well combs
pFoxo1: NB100-8192
Foxo1: CST-2880
pAkt: CST-4060S
Akt: CST-9272S
Fkbp51: WX904284
β-Actin: CST3700

Gels: 12%, 15 well combs
pFoxo1: NB100-8192
Foxo1: CST-2880
pAkt: CST-4060S
Akt: CST-9272S
Fkbp51: WX904284
β-Actin: CST3700

Gels: 12%, 15 well combs
pFoxo1: NB100-8192
Foxo1: CST-2880
pAkt: CST-4060S
Akt: CST-9272S
Fkbp51: WX904284
β-Actin: CST3700

Fig4 I

NIT-1 Nuclear protein

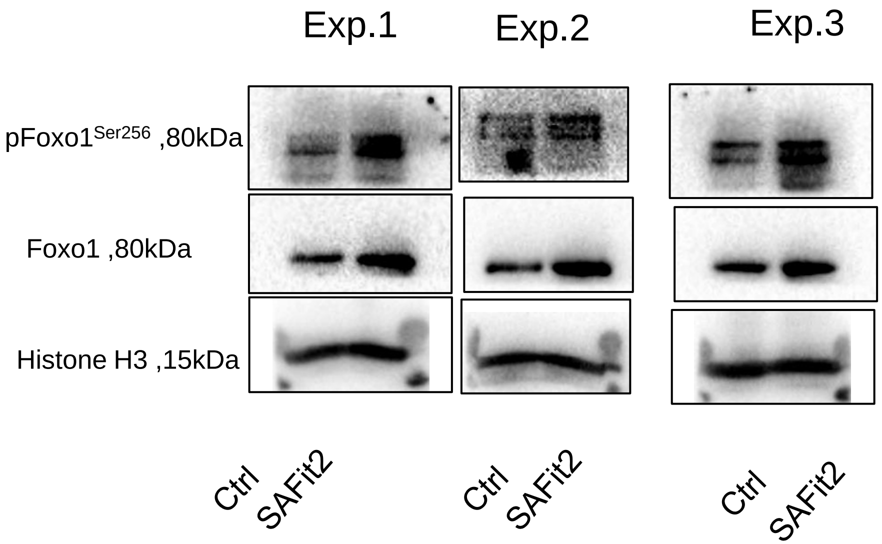

Gels: 12%, 15 well combs
pFoxo1: NB100-8192
Foxo1: CST-2880
Histone H3a : 17168-1-AP

Fig4 J

NIT-1 Cytoplasmic protein

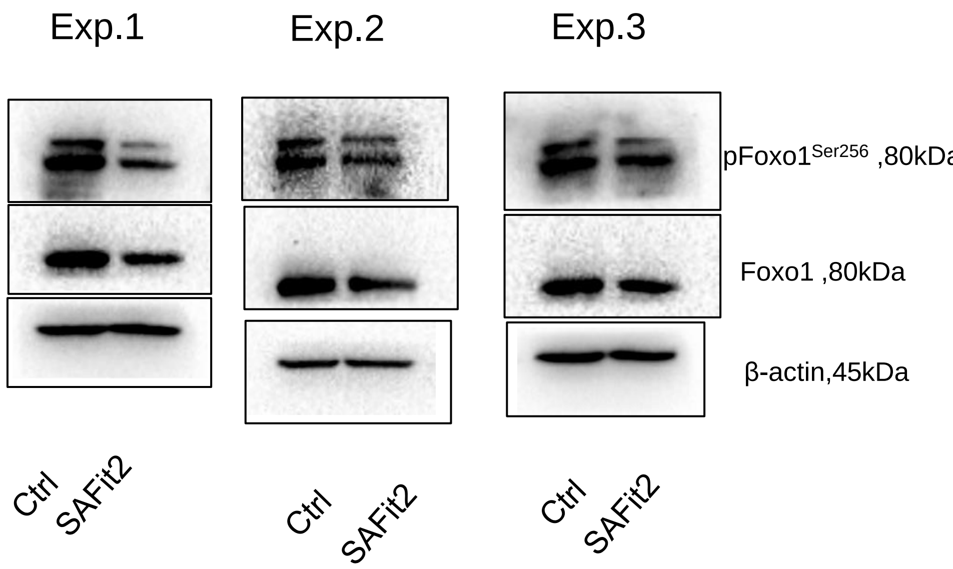

Gels: 12%, 15 well combs
pFoxo1: NB100-8192
Foxo1: CST-2880
β-actin: CST3700

Fig4 M

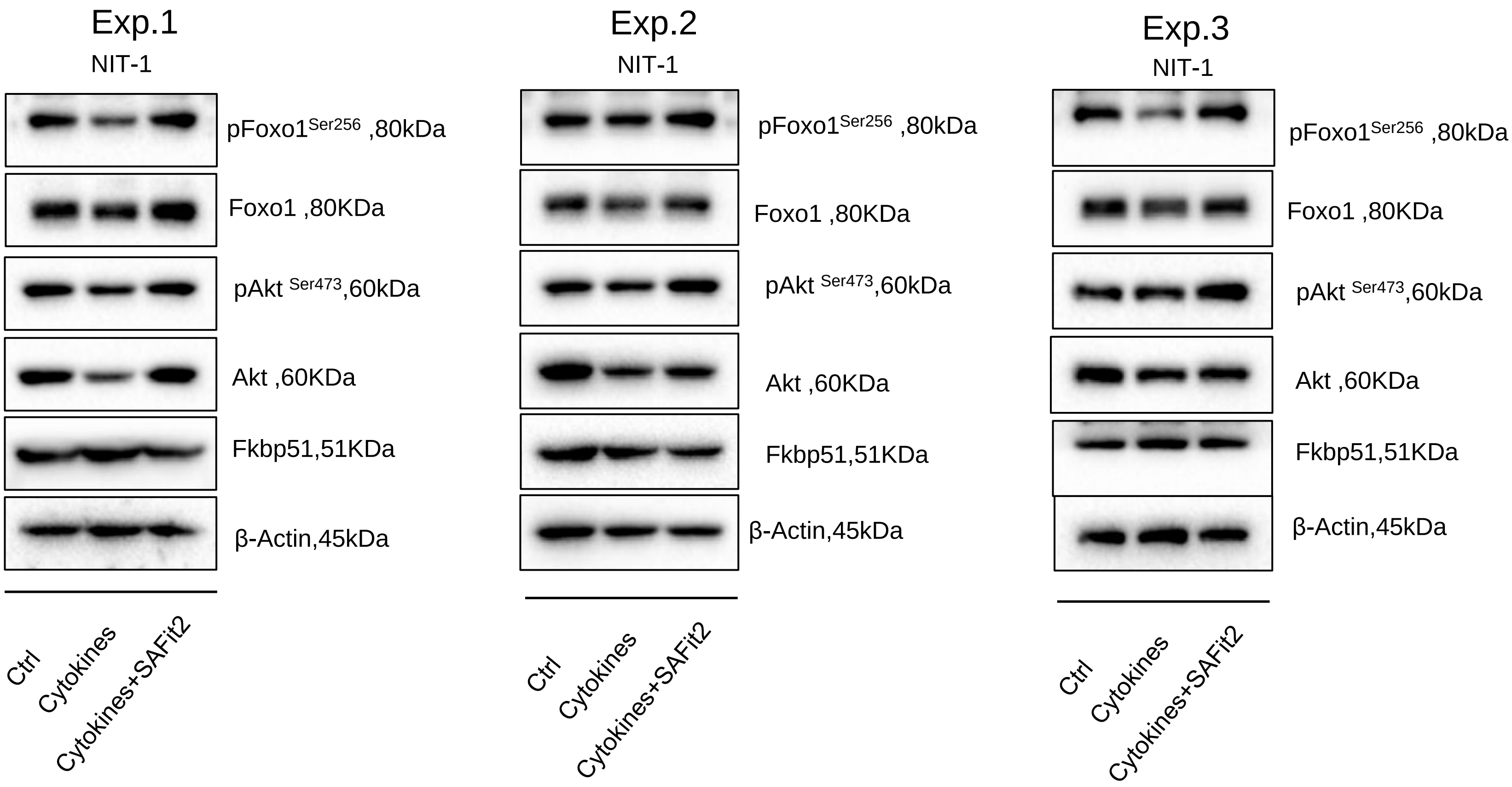

Gels: 10%, 15 well combs
pFoxo1: NB100-8192
Foxo1: CST-2880
pAkt: CST-4060S
Akt: CST-9272S
Fkbp51: WX904284
β-Actin: CST3700

Gels: 10%, 15 well combs
pFoxo1: NB100-8192
Foxo1: CST-2880
pAkt: CST-4060S
Akt: CST-9272S
Fkbp51: WX904284
β-Actin: CST3700

Gels: 10%, 15 well combs
pFoxo1: NB100-8192
Foxo1: CST-2880
pAkt: CST-4060S
Akt: CST-9272S
Fkbp51: WX904284
β-Actin: CST3700

Fig5 A

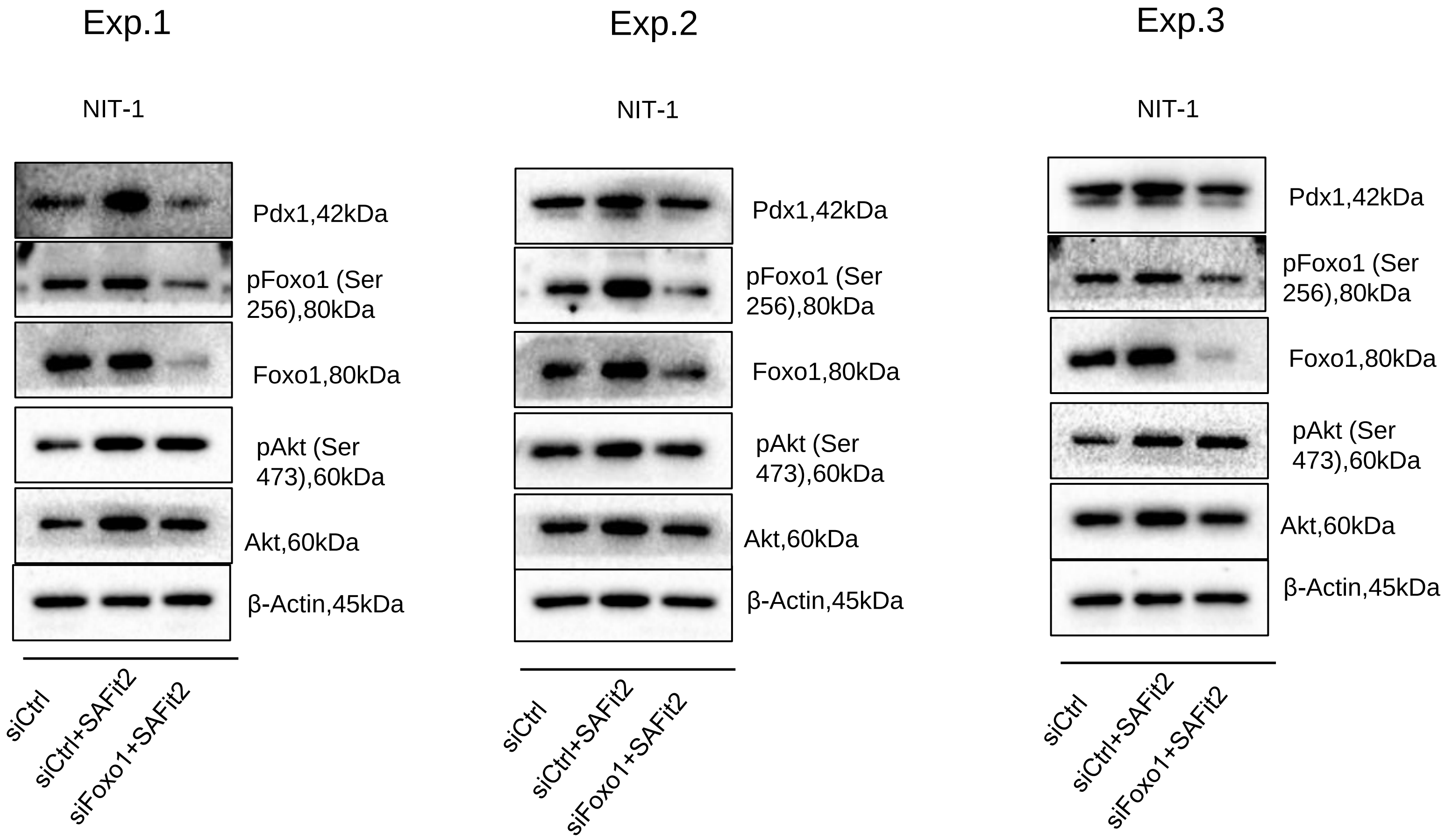

Gels: 10%, 15 well combs
pFoxo1: NB100-8192
Foxo1: CST-2880
pAkt: CST-4060S
Akt: CST-9272S
Pdx1:CST5679S
β-Actin: CST3700

Gels: 10%, 15 well combs
pFoxo1: NB100-8192
Foxo1: CST-2880
pAkt: CST-4060S
Akt: CST-9272S
Pdx1:CST5679S
β-Actin: CST3700

Gels: 10%, 15 well combs
pFoxo1: NB100-8192
Foxo1: CST-2880
pAkt: CST-4060S
Akt: CST-9272S
Pdx1:CST5679S
β-Actin: CST3700

Fig5E

Exp.1

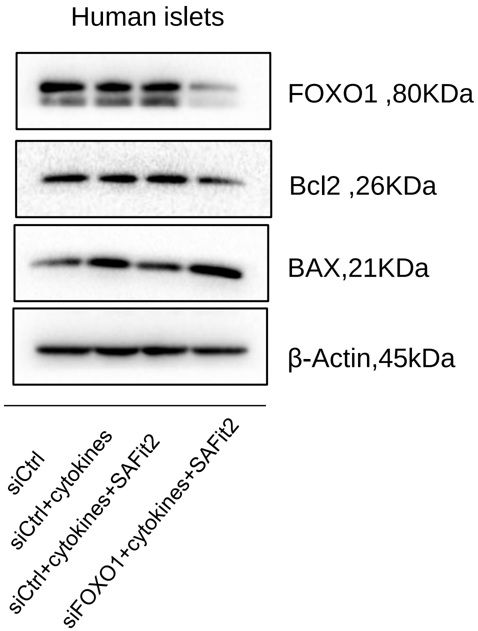

Gels: 12% 15 well combs
FOXO1: CST-2880
BAX: ab32503
BCL2: 26593-1-AP
β-Actin: CST3700

Exp.2

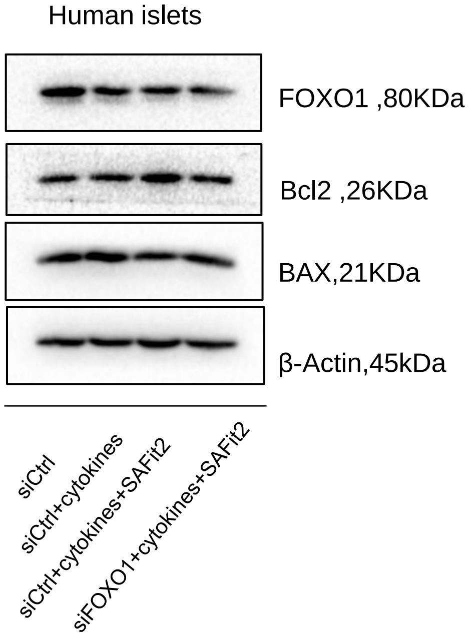

Gels: 12% 15 well combs
FOXO1: CST-2880
BAX: ab32503
BCL2: 26593-1-AP
β-Actin: CST3700

Exp.3

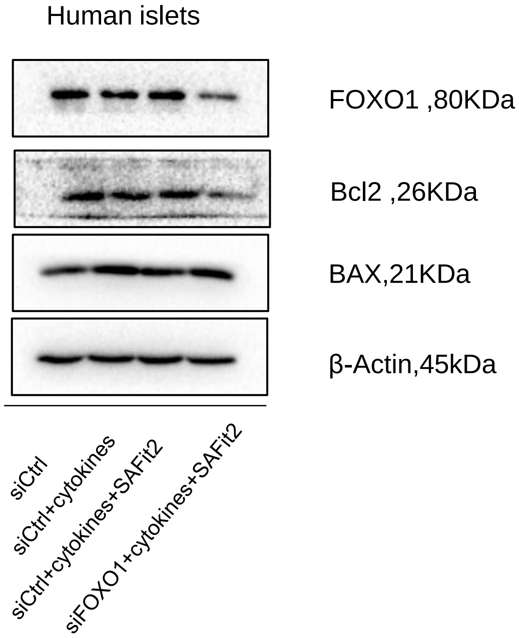

Gels: 12% 15 well combs
FOXO1: CST-2880
BAX: ab32503
BCL2: 26593-1-AP
β-Actin: CST3700

Supplementary Figure 3

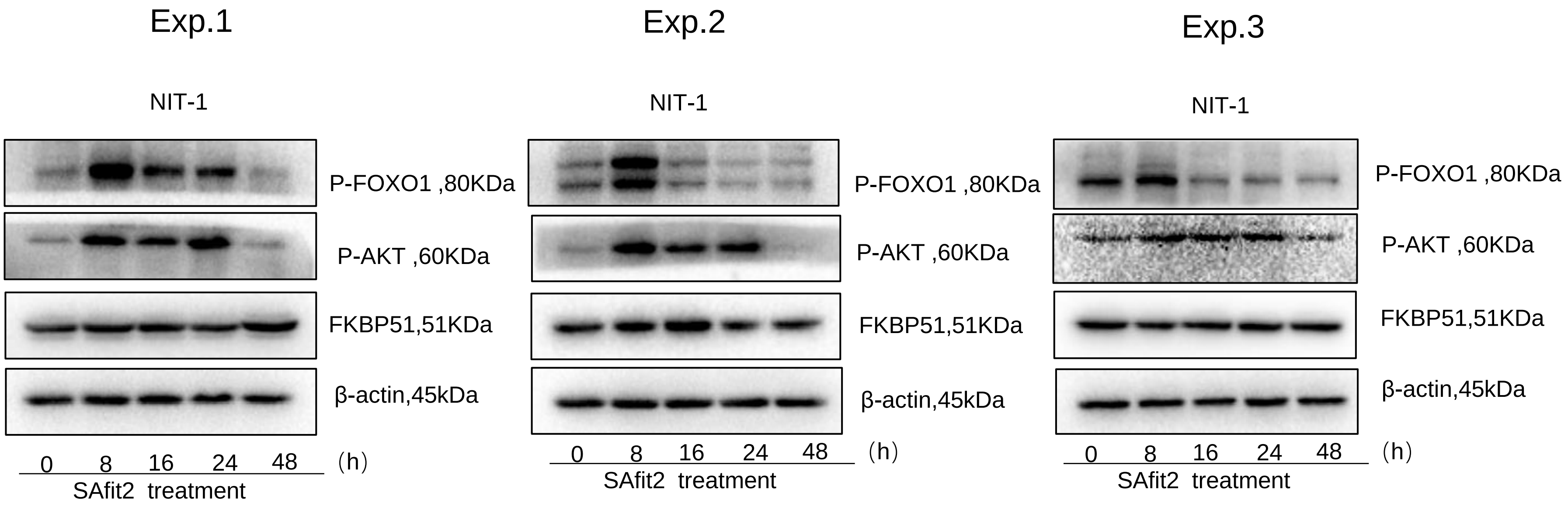

Gels: 15%, 15 well combs
P-FOXO1: NB100-8192
P-AKT: CST-4060S
FKBP51: WX904284
β-actin: CST3700

Gels: 15%, 15 well combs
P-FOXO1: NB100-8192
P-AKT: CST-4060S
FKBP51: WX904284
β-actin: CST3700

Gels: 15%, 15 well combs
P-FOXO1: NB100-8192
P-AKT: CST-4060S
FKBP51: WX904284
β-actin: CST3700

Supplementary Figure 4

Exp.1

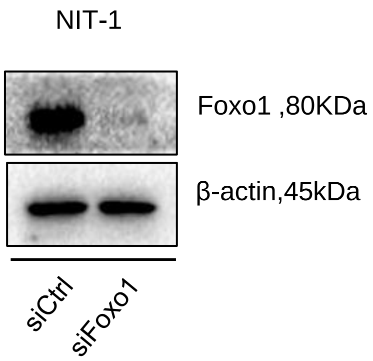

Gels: 10%, 15 well combs
Foxo1: CST-2880
β-Actin: CST3700

Exp.2

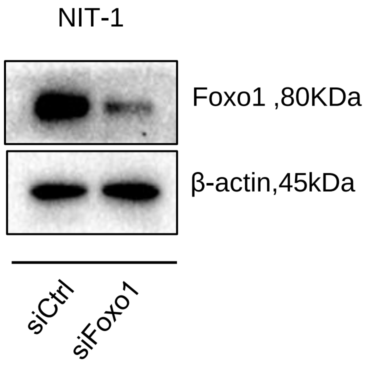

Gels: 10%, 15 well combs
Foxo1: CST-2880
β-Actin: CST3700

Exp.3

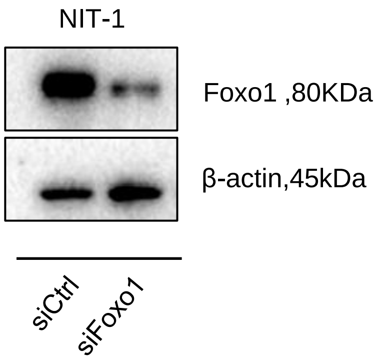

Gels: 10%, 15 well combs
Foxo1: CST-2880
β-Actin: CST3700
